# Abnormal data detection of guidance angle based on SMP-SVDD for seeker

**DOI:** 10.1038/s41598-022-05565-5

**Published:** 2022-01-27

**Authors:** Chao Liang, Dedong Cui, Zhengang Yan, Xiangyu Zhang, Qiang Luo, Jiang Hu, Xuan He

**Affiliations:** 1 School of Artificial Intelligence, Xidian University, Xi’an, 710071 China; 2 Xi’an Modern Control Technology Research Institute, Xi’an, 710065 China; 3 Xi’an Institute of Applied Optics, Xi’an, 710018 China

**Keywords:** Engineering, Mathematics and computing

## Abstract

The accuracy of the pitch angle deviation directly affects the guidance accuracy of the laser seeker. During the guidance process, the abnormal pitch angle deviation data will be produced when the seeker is affected by interference sources. In this paper, a new abnormal data detection method based on Smooth Multi-Kernel Polarization Support Vector Data Description (SMP-SVDD) is proposed. In the proposed method, the polarization value is used to determine the weight of the multi-kernel combination coefficient to obtain the multi-kernel polarization function, in which the particle swarm optimization is used to find the optimal kernels for higher detection accuracy. Besides, by using smoothing mechanism, the constrained quadratic programming problem is translated to be smooth and differentiable. Then, this problem can be solved by the conjugate gradient method, which could reduce the computational complexity. In experimental section, abundant simulation experiments were designed and the experimental results verify that the proposed SMP-SVDD method could achieve higher detection accuracy and low computational cost compared with different detection methods in different guidance stages.

## Introduction

In the actual guidance process, the laser seeker faces various interference factors, such as laser high repetition frequency interference^1^ and laser deception interference^2,3^. These interference factors will produce abnormal data about the pitch angle deviation in laser guidance data. High-frequency jamming can force the jamming signal into the time wave gate of seeker by generating a high-frequency laser signal, thus flooding the real signal and generating jamming data^4–6^. Jamming sends out a signal that is consistent with the indication signal by measuring the parameter information, such as the wavelength, frequency, and azimuth of the indicating laser, and enters the wave gate of the seeker to generate jamming data^7^. During the flight of the missile, the function of laser seeker is to measure the angle information between missile and target and the accuracy of the angle will directly affect the final hit accuracy of the missile^8^. When the laser seeker is disturbed, the abnormal seeker angle measurement data can reduce the guidance accuracy of the missile^9^. Eliminating the abnormal data from the angle measurement information of the seeker is of great significance to improve the guidance accuracy and anti-jamming ability of the missile^10^.

The elimination of interference can be regarded as the problem of outlier detection, where normal data and abnormal data can be classified to achieve the purpose of anti-interference. The abnormal interference data of laser seeker guidance can be processed by detection and classification methods. The abnormal interference data of laser seeker guidance can be processed by detection and classification. At present, there are many abnormal data detection methods, such as convolutional neural networks (CNN)^11,12^, discriminant analysis methods^13^, clustering methods^14^, support vector machine methods^15^, cascade model-aware generative (CMAG)^16^, Tradaboost methods^17^, generalized least squares methods^18^, MLP(multi-layer perceptron)^19^, probability methods^20^, local outlier factor^21^, and so on. Yuen et al.^20^ adopted a probability method for outlier detection and quantified the outlier probability of data points, considering not only the optimal values of parameters and residuals, but also the uncertainty of data. However, this method needs to give a threshold probability to judge whether the data is abnormal or not. Liu et al.^21^ proposed an outlier detection method based on local information, which combines the traditional local outlier detection method LOF with the outlier factor of uncertain information. Paola et al.^22^ proposed an adaptive distributed Bayesian method to detect outliers in data collected by wireless sensors and also considered the external constraints of these target data. However, this method needs a probability density distribution model with uncertain data, which is difficult for seeker guidance angle data. Li et al.^23^ proposed an outlier detection method based on structural scores to process high-dimensional data, which can reflect the characteristics of high-dimensional data. However, because outliers are judged by calculating the included angle of vectors and sorting the structure, this method may have a higher false detection rate for outliers with a small Euclidean distance from normal data. Yuan et al.^24^ introduced fuzzy rough set (FRSs) to deal with the problem of anomaly detection and classification of mixed attribute data, generalized the outlier detection model by FRS, and constructed a generalized outlier detection model based on fuzzy rough granules. However, this method has high time and space complexity and needs further optimization. Abid et al.^25^ adopted a density-based method to detect clusters with arbitrary shapes and outliers. However, the method based on density clustering is not suitable for data with uneven density of sample set and large cluster spacing. Support vector machine (SVM) has been introduced to solve the outlier detection problem because of its advantages in binary classification. The support vector data description SVDD is a single classification method of support vector machine, which does not need any distribution assumptions for target data, can map the original data to high-dimensional feature space, establish the smallest hypersphere containing the given data, and can detect outliers^26^. However, SVDD algorithm has high complexity, and it is difficult to select kernel functions and kernel parameters^27^.

In the actual guidance process, the pitch angle deviation data of the laser seeker in different guidance stages varies greatly nonlinearly, which makes it difficult to assume distribution. Besides, due to the limited hardware resources of the missile and the complexity of the algorithm, the above methods cannot meet the requirements of abnormal data detection of the laser seeker. Therefore, this paper proposes a smooth multi-kernel polarization support vector data description (SMP-SVDD) method to classify and detect the pitch angle deviation data. Compared with single-kernel kernel function, multi-kernel function can adapt to data with different nonlinear characteristics and improve the detection accuracy of the algorithm. However, because the SVDD algorithm needs to solve quadratic programming problems, the complexity of the algorithm is high, and multi-kernel will also increase the complexity of the algorithm to a certain extent, thus these factors will increase the resource consumption of the onboard system. Therefore, the proposed method also introduces the smoothing function to reduce the complexity of the algorithm, by transforming the constrained quadratic programming problem into an unconstrained differentiable optimization problem which can be solved by conjugate gradient method. However, because the nonlinear characteristics of data in different stages are quite different, this method adopts a multi-stage method to construct the detection model, and adopts the particle swarm optimization method to determine the optimal kernel function and kernel parameters in each stage. Experiments show that this method is effective in dealing with outliers of the seeker pitch angle deviation data.

The rest of this paper is organized as follows. In the second part, the theoretical calculation and analysis of smooth multi-kernel polarization support vector data description algorithm are given, including classical SVDD algorithm, multi-core polarization SVDD algorithm, smooth multi-kernel polarization SVDD algorithm, optimal selection of kernel parameters and algorithm complexity analysis. In the third part, through simulation experiments, we verify the detection performance of the proposed method both on detection accuracy and computational cost. Finally, a conclusion of this work is given.

## Smooth multi-kernel polarization support vector data description

### Support vector data description (SVDD)

The basic idea of the support vector data description is to map the normal data to the high-dimensional feature space, construct a minimum hypersphere to describe the data, contain all the normal data, and eliminate the outliers from the outliers^26^. The goal of SVDD is to find a minimum radius to distinguish outliers form normal data.

Take the pitch angle deviation data of the laser seeker as the training sample $\{ {{\varvec{\uptheta}}}_{i} ,i = 1, \ldots m\}$, ${{\varvec{\uptheta}}}_{i}$ contains the normal pitch angle deviation data and the disturbed data, and these data are marked. We described the data set, the simplest model is to use a hypersphere to simulate the distribution area of the positive sample.

SVDD is the non-linear transformation $\Phi$ mapping of the training sample data ${{\varvec{\uptheta}}}_{i}$ to find the smallest volume hypersphere $\Omega = ({{\varvec{\upalpha}}},R)$ that surrounds all or most of the positive samples, where ${{\varvec{\upalpha}}}$ represents the hypersphere center and R represents the hypersphere radius. Mathematically, it can be expressed as the following formula:

1 $$ \begin{aligned} & \mathop {\min }\limits_{{R,{{\varvec{\upalpha}}},{{\varvec{\upxi}}}}} F(R,{{\varvec{\upalpha}}},{{\varvec{\upxi}}}) = R^{2} + C\sum\limits_{i = 1}^{n} {\xi_{i} } \\ & s.t. \, \left\| {\Phi ({{\varvec{\uptheta}}}_{i} ) - {{\varvec{\upalpha}}}} \right\|^{2} \le R^{2} + \xi_{i} , \, \xi_{i} > 0. \\ \end{aligned} $$

The center ${{\varvec{\upalpha}}}$ of the hypersphere can be expressed as a Lagrangian multiplier^27^:

2 $$ {{\varvec{\upalpha}}} = \sum\limits_{i = 1}^{n} {\alpha_{i} \Phi ({{\varvec{\uptheta}}}_{i} )} . $$

By constructing a Lagrange function, the original problem can be transformed into the following problem:

3 $$ \begin{aligned} & \max \sum\limits_{i} {\alpha_{i} K({{\varvec{\uptheta}}}_{i} \cdot {{\varvec{\uptheta}}}_{i} ) - \sum\limits_{i,j} {\alpha_{i} \alpha_{j} K({{\varvec{\uptheta}}}_{i} \cdot {{\varvec{\uptheta}}}_{j} )} } \\ & s.t. \, \sum\limits_{i} {\alpha_{i} } = 1, \, 0 \le \alpha_{i} \le C, \\ \end{aligned} $$

where $K({{\varvec{\uptheta}}}_{i} \cdot {{\varvec{\uptheta}}}_{j} ) = \langle \Phi ({{\varvec{\uptheta}}}_{i} ),\Phi ({{\varvec{\uptheta}}}_{j} )\rangle$ is the kernel function.

By solving the linear constrained quadratic optimization problem mentioned above, $\alpha_{i}$ can be obtained. Only when $\alpha_{i} > 0$, the sample point ${{\varvec{\uptheta}}}_{i}$ of the seeker pitch angle deviation data affects the center of the hypersphere, and the corresponding sample point is called the support vector. The radius of the hypersphere can be expressed as

4 $$ R^{2} = K({{\varvec{\uptheta}}}_{k} \cdot {{\varvec{\uptheta}}}_{i} ) - 2\sum\limits_{i} {\alpha_{i} } K({{\varvec{\uptheta}}}_{k} \cdot {{\varvec{\uptheta}}}_{i} ) + \sum\limits_{i,j} {\alpha_{i} \alpha_{j} K({{\varvec{\uptheta}}}_{i} \cdot {{\varvec{\uptheta}}}_{j} )} . $$

The distance from the test data sample ${{\varvec{\uptheta}}}_{i}^{^{\prime}}$ to the center of the hypersphere is expressed as

5 $$ \left\| {{{\varvec{\uptheta}}}_{i}^{^{\prime}} - {{\varvec{\upalpha}}}} \right\|^{2} = K({{\varvec{\uptheta}}}_{i}^{^{\prime}} \cdot {{\varvec{\uptheta}}}_{i}^{^{\prime}} ) - 2\sum\limits_{i} {\alpha_{i} } K({{\varvec{\uptheta}}}_{i}^{^{\prime}} \cdot {{\varvec{\uptheta}}}_{i} ) + \sum\limits_{i,j} {\alpha_{i} \alpha_{j} K({{\varvec{\uptheta}}}_{i} \cdot {{\varvec{\uptheta}}}_{j} )} , $$

where ${{\varvec{\uptheta}}}_{k} \in SVs$, $SVs$ is the support vector set. If $\left\| {{{\varvec{\uptheta}}}_{i}^{^{\prime}} - {{\varvec{\upalpha}}}} \right\|^{2} \le R^{2}$, then ${{\varvec{\uptheta}}}_{i}^{^{\prime}}$ is the pitch angle deviation data without interference; otherwise, it is the interference data.

### Multi-kernel polarization SVDD (MP-SVDD)

The pitch angle deviation data of the laser seeker will show different nonlinear characteristics in different stages. Therefore, when using the SVDD model, compared with a single-kernel function, multi-kernel function has a stronger classification ability and better flexibility for data in different guidance stages. However, in the process of multi-kernel combination, numerous combination weight parameters will be artificially introduced, which will make it difficult to find the best parameters, and it is easy to have a dimension disaster and local extremum problems when searching for the best parameters.

Polarization can reflect the similarity between a kernel function and an ideal kernel matrix. The same kind of data is close to each other, while different kinds of data are far away from each other, and the combination relationship between different kernels can be determined^28,29^. If there is a clear correspondence between the nuclear data points and the labeled values, the classification process will become easier. Suppose that the training data set is $\{ x^{(i)} ,y^{(i)} ,i = 1,...M\}$, *y* is the labeled data, $y^{(i)} \in \{ - 1, + 1\}$, the polarization nucleus is defined as

6 $$ K_{v} = \frac{1}{{M^{2} }}\sum\limits_{i = 1}^{M} {\sum\limits_{j = 1}^{M} {k_{v} (x^{(i)} ,x^{(j)} )} } y^{(i)} y^{(j)} . $$

The greater the contribution rate of the kernel function to the correct classification of the sample, the greater the corresponding $K_{v}^{(i)}$ value would be. Therefore, in the multi-kernel learning process, the nuclear polarization value can be used to determine the weight of the combination coefficient. The specific expression for determining the weight coefficient is as follows:

7 $$ \lambda_{i} = \frac{{K_{v}^{(i)} }}{{\sum\nolimits_{i = 1}^{n} {K_{v}^{(i)} } }}. $$

In this work, we chose the following basic kernel functions: Gaussian kernel function, Laplace kernel function, and exponential kernel function. We can combine the following polynuclear polarization functions as follows:

8 $$ \left\{ \begin{gathered} K_{GL} = \lambda_{G} \cdot K_{G} + \lambda_{L} \cdot K_{L} \hfill \\ K_{GE} = \lambda_{G} \cdot K_{G} + \lambda_{E} \cdot K_{E} \hfill \\ K_{LE} = \lambda_{L} \cdot K_{L} + \lambda_{E} \cdot K_{E} \hfill \\ K_{GLE} = \lambda_{G} \cdot K_{G} + \lambda_{L} \cdot K_{L} + \lambda_{E} \cdot K_{E} . \hfill \\ \end{gathered} \right. $$

Among them, $K_{G}$,$K_{L}$,$K_{E}$ are the Gaussian kernel function, Laplace kernel function, and exponential kernel function. $K_{G}$,$K_{L}$,$K_{E}$ are the combined multi-kernel polarization function. Using a multi-kernel polarization kernel function in SVDD, the following dual optimization form is obtained:

9 $$ \begin{aligned} & \max \sum\limits_{i} {\alpha_{i} K_{{{\text{mp}}}} ({{\varvec{\uptheta}}}_{i} \cdot {{\varvec{\uptheta}}}_{i} ) - \sum\limits_{i,j} {\alpha_{i} \alpha_{j} K_{{{\text{mp}}}} ({{\varvec{\uptheta}}}_{i} \cdot {{\varvec{\uptheta}}}_{j} )} } \\ & s.t. \, \sum\limits_{i} {\alpha_{i} } = 1, \, 0 \le \alpha_{i} \le C \, \forall i. \\ \end{aligned} $$

Among them, $K_{{\text{m - p}}}$ is a multi-kernel polarized kernel function, including four types of kernel functions: $K_{GL}$,$K_{GE}$,$K_{LE}$, and $K_{GLE}$.

### Smooth MP-SVDD

Because MP-SVDD is still an optimization problem in the form of quadratic programming, it cannot be directly converted into an unconstrained differentiable function for optimization. This leads to high algorithm complexity in the process of seeker angle data training, and the training time will increase geometrically with the increase of data. Inspired by the smoothing function, the MP-SVDD model is smoothed and transformed into a differentiable unconstrained optimization problem, and the conjugate gradient method is used to find the optimal solution.

The smooth function can be obtained by integrating the sigmoid function^30^.

10 $$ p_{\tau } (x) = x + \frac{1}{\alpha }\ln (1 + e^{ - \tau x} ). $$

Let $L(\alpha ) = K_{mp} ({{\varvec{\uptheta}}}_{i} \cdot {{\varvec{\uptheta}}}_{i} ) - 2\sum\nolimits_{i} {\alpha_{i} } K_{mp} ({{\varvec{\uptheta}}}_{i} \cdot {{\varvec{\uptheta}}}_{j} ) + \sum\nolimits_{j,k} {\alpha_{j} \alpha_{k} K_{mp} ({{\varvec{\uptheta}}}_{j} \cdot {{\varvec{\uptheta}}}_{k} )}$, then the aforementioned constrained quadratic programming optimization problem can be transformed into a differentiable $F_{\tau }$ function:

11 $$ F_{\tau } (R,\alpha ) = R^{2} + C\sum\limits_{i = 1}^{n} {p_{\tau } \left( {L(\alpha ) - R^{2} } \right)} . $$

The partial derivative of the R and $\alpha$ variables in the formula can be obtained as follows:

12 $$ \frac{{\partial F_{\tau } }}{{\partial R^{2} }}{ = }1 - C\sum\limits_{i = 1}^{n} {p_{\tau }^{^{\prime}} \left( {L(\alpha ) - R^{2} } \right)} . $$

13 $$ \frac{{\partial F_{\tau } }}{\partial \alpha } = C \cdot L^{^{\prime}} (\alpha )\sum\limits_{i = 1}^{n} {p_{\tau }^{^{\prime}} \left( {L(\alpha ) - R^{2} } \right)} . $$

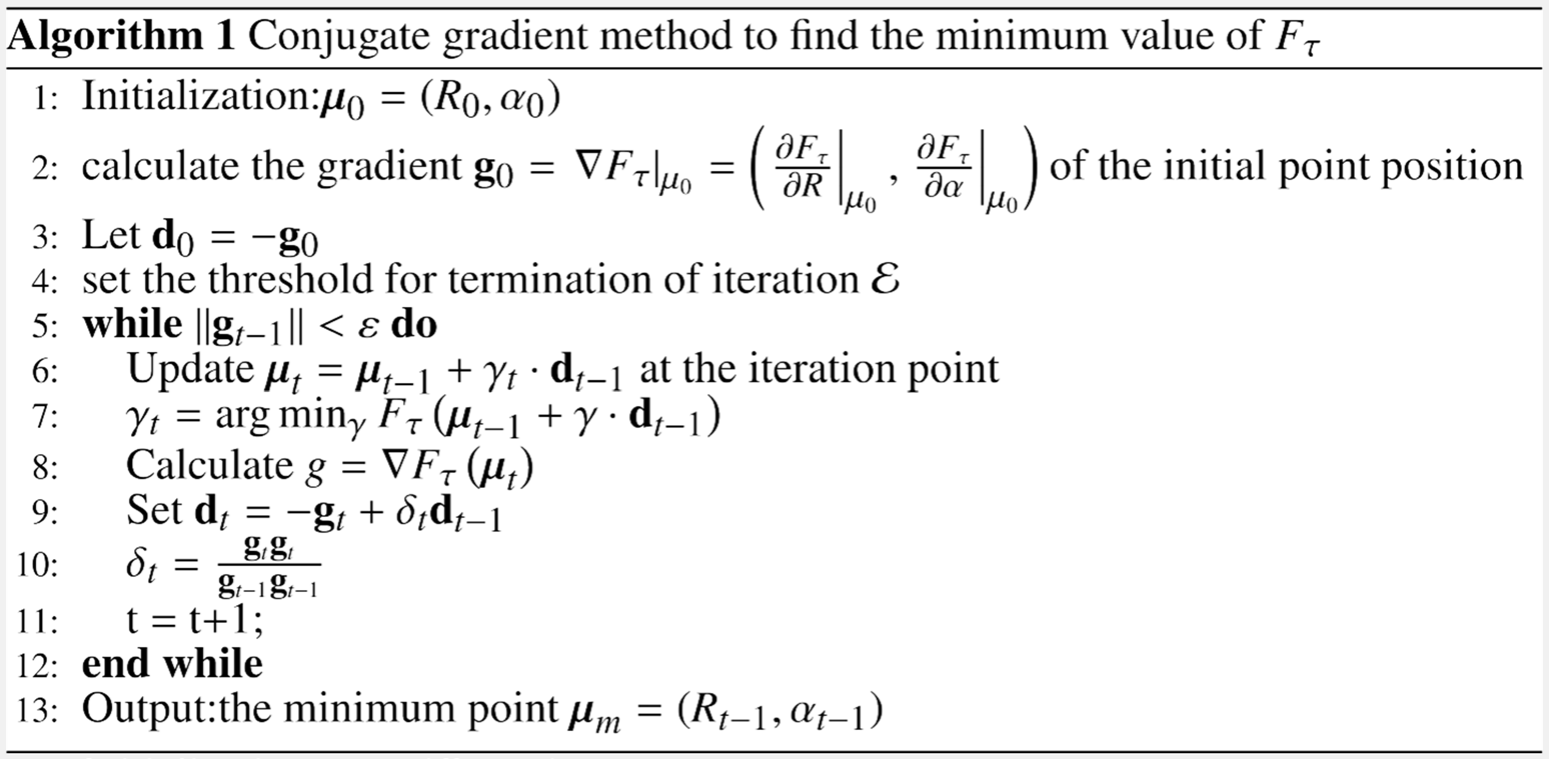


Compared with the constrained quadratic programming problem, the conjugate gradient method mentioned above avoids the complicated operations, such as solving linear matrix equations, by which the complexity of the algorithm can be reduced.

### Optimal selection of nuclear parameters

The pitch angle deviation data of the seeker has different nonlinear characteristics at different stages, and the classification accuracy of pitch angle deviation data is different with different kernel function parameters and different linear combinations. Therefore, the particle swarm optimization algorithm is adopted in this paper, and different kernel function parameters are adopted for different guidance stages to obtain the optimal multi-kernel function and penalty factor.
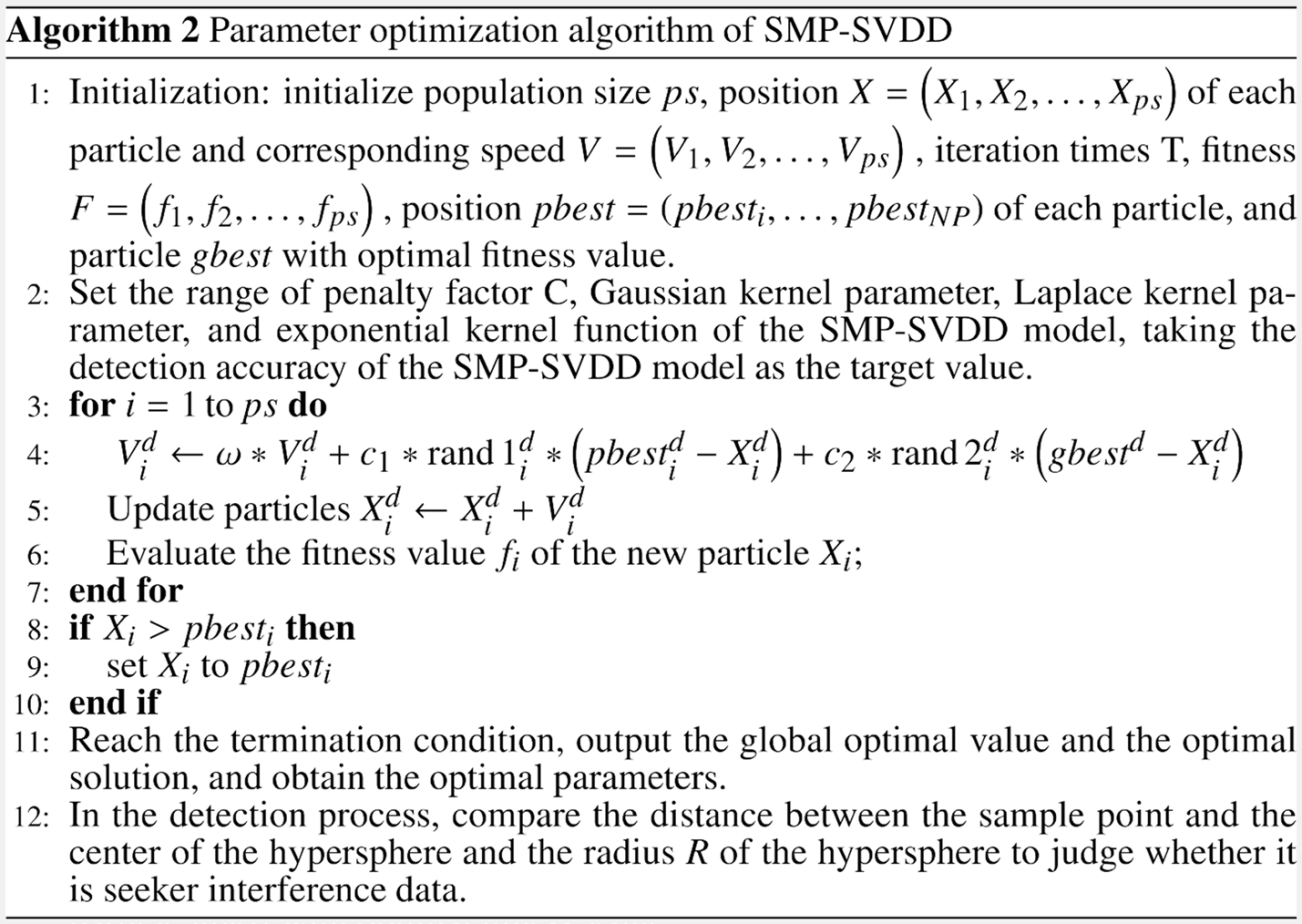


Particle Swarm Optimization (PSO) is a heuristic evolutionary computation technique, which initializes a group of particles and iterates to find the optimal solution. Particle Swarm Optimization is widely used in target optimization^31,32^, neural network training^33,34^, and so on. The PSO method defines a fitness function according to the objective function, and every particle is updated by speed and position in the iterative a process of optimization. Every particle will determine a local optimal solution *ipbest*, and the optimal solution found by the whole population is called global optimal solution *gbest*. The PSO algorithm adaptively updates the speed and position information of particles based on the good past experience.

**Figure 1:**
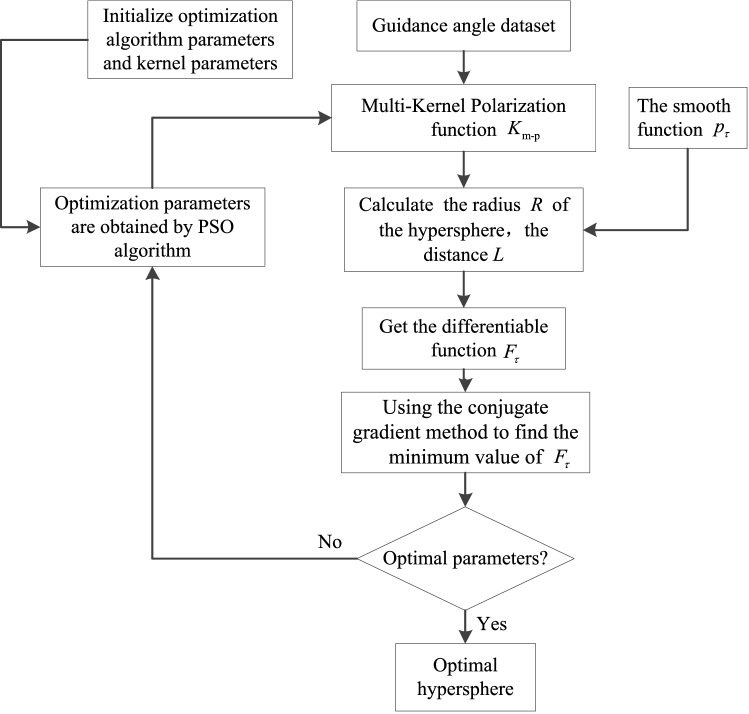
Flow chart of the proposed method in this paper.

The process of PSO algorithm to optimize the SMP-SVDD model is shown in Algorithm 2. Among them, $\omega$ is the inertia weight, which is used to measure the search ability of the particle swarm optimization algorithm, $c_{1}$ is the individual learning factor, and $c_{2}$ is the group learning factor. As shown in Fig. 1, the parameters in SMP-SVDD are optimized by PSO algorithm.

### Complexity analysis

Assuming that there are *N* data in the whole guidance phase, the time complexity of the classical SVDD algorithm^27^ is $O(N^{3} )$, and that of the SA-SVDD algorithm is $O(N^{2} )$. In the SMP-SVDD model, the time complexity of the polarization kernel function $K_{v} = \frac{1}{{M^{2} }}\sum\nolimits_{i = 1}^{M} {\sum\nolimits_{j = 1}^{M} {k_{v} (x^{(i)} ,x^{(j)} )} } y^{(i)} y^{(j)}$ is $O(N^{2} )$ after the multi-kernel polarization function is calculated, the smoothing process is performed, and the conjugate gradient is used to solve the problem, in which the most complicated operation is $\sum\nolimits_{j,k} {\alpha_{j} \alpha_{k} K_{mp} ({{\varvec{\uptheta}}}_{j} \cdot {{\varvec{\uptheta}}}_{k} )}$ and the complexity is $O(N^{2} )$. Therefore, the computational complexity of SMP-SVDD is $O(N^{2} )$.

However, the characteristics of pitch angle deviation data are quite different in each guidance stage. If the data of the whole guidance stage is trained at one time, it will not only be difficult to ensure the accuracy of data detection, but the computational complexity will also increase geometrically because of the increase in data volume in the whole process. If the entire guidance process is divided into n guidance stages according to the characteristics of different stages, the data volume of each stage is $\left\{ {\frac{N}{{n_{1} }},\frac{N}{{n_{2} }},...,\frac{N}{{n_{i} }},...\frac{N}{{n_{n} }}} \right\}$. Because the time complexity and the data volume are quadratic, $T(N) > \sum\nolimits_{i = 1}^{n} {T\left( {\frac{N}{{n_{i} }}} \right)}$, where $T( \cdot )$ is the calculation operation of the algorithm time.

## Simulation experiments

### Evaluation indexes

In this paper, the accuracy rate, recall rate (TPR), false positive rate (FPR), true negative rate (TNR), and false negative rate (FNR) are used to evaluate the detection performance of the model. The higher the accuracy and recall rate, the better the performance of the model. The statistical result of sample classification is shown in Table 1.

**Table 1:** Classification of samples.

Actual situation	Testing result	
	Positive class	Negative class
Positive class	TP (real positive)	FN (false negative)
Negative class	FP (false positive)	TN (true negative)

The calculation formulas of evaluation indexes are as follows:

15 $$ \left\{ \begin{gathered} Accuracy = \frac{TP + TN}{{(TP + FN) \, + \, (FP + TN)}},TPR = \frac{TP}{{TP + FN}} \hfill \\ TNR = \frac{TN}{{FP + TN}},FPR = \frac{FP}{{FP + TN}},FNR = \frac{FN}{{TP + FN}}. \hfill \\ \end{gathered} \right. $$

### Experimental results of comparing algorithms

In this section, the experiments simulate the laser guided missile attacking the ground target. The whole trajectory simulation range is 8 km. When the seeker is 5 km away from the target, it starts to guide. When it is 3 to 5 km away from the target, it is the initial guidance stage, wherein the seeker is in the state of searching for the target and tracking it, the intermediate guidance process is 1.5 to 3 km away from the target, and the final guidance stage is 0 to 1.5 km away. Trajectory simulation is conducted under the conditions of no interference and laser decoy interference (4 to 2.5 km with interference), and the pitch angle deviation data set during laser seeker guidance is obtained. The specific conditions of the data set are shown in Table 2. In this paper, MATLAB 2018b is used to run on PC and the CPU is an AMD Ryzen 7 5800H 3.2GHz with 16GB RAM.

**Table 2:** Experimental data set.

Guidance phase	Normal data	Abnormal data
Initial stage	343	172
Intermediate stage	269	132
Final stage	328	61
Overall process	949	346

According to the experimental dataset obtained from ballistic simulation, we used SVDD, SA-SVDD, and SMP-SVDD to detect the outliers of pitch angle deviation data in the whole guidance process. Through setting optimization parameters, the particle population size is 60, the maximum number of iterations is 1000, the range of penalty factor is [0 1], the range of kernel parameters of Gaussian kernel function is [0.1 10], the range of kernel parameters of Laplace kernel function is [0.1 10], and the range of kernel parameters of exponential kernel function is [0.1 10]. The comparison results can be obtained through optimization, as shown in Table 3

**Table 3:** Comparison of outlier detection indexes of pitch angle deviation in the whole guidance stage.

Model	Kernel function	TPR (%)	TNR (%)	FPR (%)	FNR (%)	Accuracy (%)
SVDD	Gauss	93.72	80.34	19.66	6.28	90.67
	Laplacian	92.37	85.96	14.04	7.63	91.37
	Exponential	93.12	83.15	16.85	6.88	90.28
SA-SVDD	Gauss	91.52	78.44	21.56	8.48	90.34
	Laplacian	91.35	83.56	16.44	8.65	90.21
	Exponential	90.22	80.35	19.65	9.78	89.89
SMP-SVDD	$K_{GL}$	99.68	82.30	17.70	0.32	94.91
	$K_{GE}$	**99.79**	83.15	16.85	0.21	95.22
	$K_{LE}$	99.04	85.39	14.61	0.96	95.29
	$K_{GLE}$	97.77	**93.26**	6.74	2.23	**96.53**

Significant values are in bold.

When compared to SVDD and SA-SVDD, the SMP-SVDD model used in this paper has higher accuracy in data classification and detection, and the highest detection accuracy is obtained when the $K_{GLE}$ kernel function is used. Comparing the TPR and TNR indicators, the detection accuracy of SMP-SVDD is improved, and the false detection rate is reduced. This shows that after the multi-kernel polarization method is used to process the kernel function, the algorithm model has adapted to the linear and non-linear changes of the data during the entire guidance process, and the classification ability and detection accuracy of the model can be improved.

### Experimental results of different kernel functions

**Figure 2:**
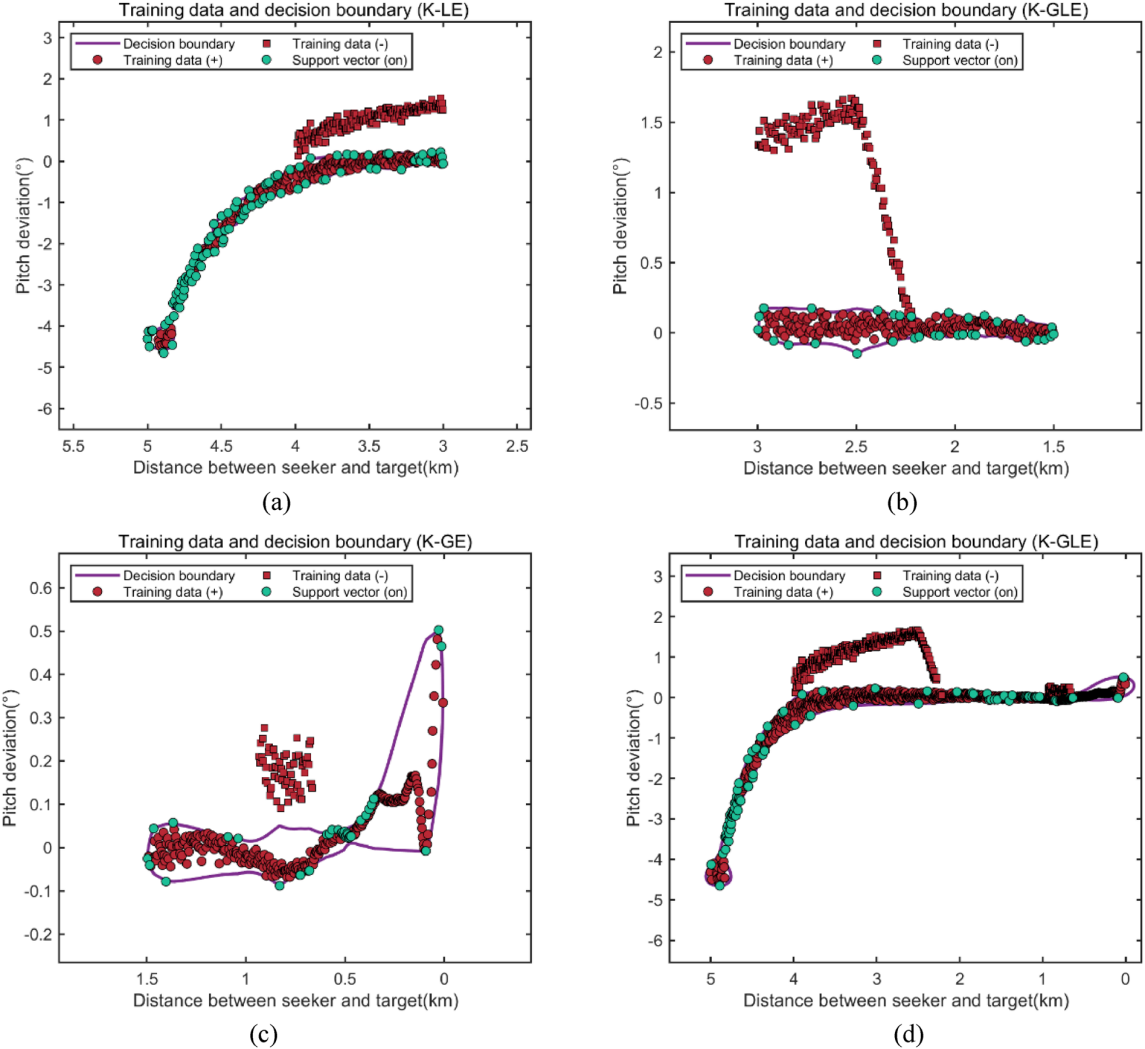
Optimal classification results of outlier detection in different guidance stages. (**a**) Outlier detection in the initial stage of guidance, (**b**) outlier detection in the intermediate stage of guidance, (**c**) Outlier detection in the final stage of guidance, (**d**) Outlier detection in the whole stage of guidance.

According to the data in different stages, the SMP-SVDD model is used for detection, and particle swarm optimization is used to find the optimal parameters of different polarization kernel functions in different guidance stages, as shown in Table 4. We can obtain the optimal kernel selection of each stage, and the classification result diagram of training data and support vector through the optimal polarization kernel function SVDD of each stage is shown in Fig. 2. Because the nonlinear characteristics of data will significantly change in different guidance stages, the outlier interference points of data in different guidance stages are detected and classified in this paper.

**Table 4:** Optimal detection results of SMP-SVDD using different polarization kernel functions at different stages.

Guidance phase	Polynuclear polarization function	Optimal kernel parameter			Number of support vectors	Accuracy of detection (%)
		Gauss	Laplace	Index		
Initial stage	$K_{GL}$	0.51	0.32	–	95	92.04
	$K_{GE}$	0.62	–	0.54	99	93.20
	$K_{LE}$	–	0.81	0.83	98	**96.70**
	$K_{GLE}$	0.71	0.52	0.61	92	94.76
Intermediate stage	$K_{GL}$	0.75	0.43	–	30	98.00
	$K_{GE}$	0.41	–	0.42	36	96.76
	$K_{LE}$	–	0.52	0.82	32	97.76
	$K_{GLE}$	0.50	0.52	0.78	27	**98.75**
Final stage	$K_{GL}$	0.51	0.54	–	35	99.09
	$K_{GE}$	0.60	–	0.80	37	**99.49**
	$K_{LE}$	–	0.59	0.81	30	95.12
	$K_{GLE}$	0.50	0.51	0.79	39	98.97
Whole stage	$K_{GL}$	3.70	3.71	–	40	94.91
	$K_{GE}$	3.00	–	0.50	31	95.22
	$K_{LE}$	–	2.90	1.90	180	95.29
	$K_{GLE}$	0.52	2.70	1.90	120	**96.53**

Significant values are in bold.

According to the results above, when using the SMP-SVDD model to detect outlier data points in the initial stage of guidance, the $K_{LE}$ polarization multi-kernel function can be used to obtain the highest detection accuracy of 96.70%. In the intermediate stage of guidance, the highest detection accuracy of 98.75% can be obtained by using the $K_{GLE}$ kernel. In the final stage of guidance, the highest detection accuracy of 99.49% can be obtained by using the $K_{GE}$ polarization multi-kernel function. By contrast, if the same multi-kernel polarization kernel function is used for the detection of outlier interference points in the entire stage, the detection accuracy is lower than that of the multi-kernel function used in stages. Compared with the detection in three different stages using different multi-kernel polarization functions, the detection accuracy of the whole stage is reduced by 2.26%, 4.31%, and 5.05%, respectively, compared with the optimized staged accuracy. Therefore, in different stages of guidance, using different polarization multi-kernel functions can achieve higher detection accuracy.

### Experimental results about time cost

According to the guidance angle data of laser seeker in the whole guidance stage and different guidance stages, under the hardware and software environment described in this section, the time of single sample training of SVDD, SA-SVDD and SMP-SVDD is compared to verify the time complexity of different algorithms.

As shown in Table 5, from the comparison of the results, the training time of SMP-SVDD is lower than that of the SVDD algorithm because the SMP-SVDD uses a conjugate gradient method to solve the minimum value, which reduces the complexity of the algorithm. Compared with SA-SVDD, SMP-SVDD uses multi-kernel function, in which its training time is slightly higher than the SA-SVDD algorithm. However, if the multi-stage training method is adopted, the data of different stages of guidance will be trained separately, which will not only improve the detection rate, but may also reduce the overall training time.

**Table 5:** Comparison of training time of different methods.

Algorithm	Processing mode		Number of detected data	Training time (s)
SVDD	Whole stage		1295	4.103
	Multi-stage processing	Initial stage	515	0.619
		Intermediate stage	401	0.391
		Final stage	389	0.339
SA-SVDD	Whole stage		1295	0.318
	Multi-stage processing	Initial stage	515	0.125
		Intermediate stage	401	0.101
		Final stage	389	0.095
SMP-SVDD	Whole stage		1295	0.326
	Multi-stage processing	Initial stage	515	0.131
		Intermediate stage	401	0.114
		Final stage	389	0.107

## Conclusion

In this paper, a SMP-SVDD method is proposed to detect the abnormal data of seeker interference and the particle swarm optimization algorithm is used to get the best kernel parameters. (1) Compared with SVDD and SA-SVDD, SMP-SVDD has better detection accuracy and higher detection accuracy. (2) The smoothing function is introduced to transform the constrained quadratic programming problem into a differentiable unconstrained problem and the conjugate gradient solution can reduce the complexity of the algorithm. Compared with SA-SVDD, the detection accuracy is improved and the calculation efficiency is slightly reduced, but the difference is not large. (3) Various polarization multi-kernel functions can be used in different guidance stages. Compared with using a polarization multi-kernel function in the whole guidance stage, this processing mode has better detection and classification performance and it improves the overall data training efficiency. The improvement in the detection performance of the seeker's interference anomaly data meant that seekers will have higher intelligent processing abilities and anti-interference performance. In the future, we will conduct further in-depth research on the detection and recognition of interference data in view of the improvement of the seeker's anti-interference performance.
